# Risk of second primary malignancies among patients with prostate cancer: A population-based cohort study

**DOI:** 10.1371/journal.pone.0175217

**Published:** 2017-04-06

**Authors:** Chao-Yueh Fan, Wen-Yen Huang, Chun-Shu Lin, Yu-Fu Su, Cheng-Hsiang Lo, Chih-Cheng Tsao, Ming-Yueh Liu, Cheng-Li Lin, Chia-Hung Kao

**Affiliations:** 1 Department of Radiation Oncology, Tri-Service General Hospital, National Defense Medical Center, Taipei, Taiwan; 2 Management Office for Health Data, China Medical University Hospital, Taichung, Taiwan; 3 College of Medicine, China Medical University, Taichung, Taiwan; 4 Graduate Institute of Clinical Medical Science, School of Medicine, College of Medicine, China Medical University, Taichung, Taiwan; 5 Department of Nuclear Medicine and PET Center, China Medical University Hospital, Taichung, Taiwan; 6 Department of Bioinformatics and Medical Engineering, Asia University, Taichung, Taiwan; Thomas Jefferson University, UNITED STATES

## Abstract

**Purpose:**

The rising incidence and life expectancy associated with prostate cancer (PCa) has led to increasing interest in predicting the risk of second primary malignancies (SPMs) among PCa survivors, although data regarding SPMs after PCa are controversial.

**Methods:**

We identified 30,964 patients from the National Health Insurance Research Database in Taiwan who had newly diagnosed PCa between 2000 and 2010. Each patient was randomly frequency-matched with an individual without PCa, based on age, comorbidity, and index year. Competing-risks regression models were used to estimate subhazard ratios (SHRs) of SPMs development associated with PCa. The Bonferroni adjustment was used in multiple comparisons.

**Results:**

Men with PCa had a trend of lower risk of developing overall SPMs compared to those without PCa (adjusted SHR = 0.94, 99.72% confidence interval [CI] = 0.89–1.00, p = 0.06). The risks of lung and liver cancer were significantly lower. In contrast, these patients had a significantly higher risk of thyroid cancer. There is a trend for a higher risk of developing SPMs in the urinary bladder and rectum/anus. Further analyses indicated that PCa patients who received radiation therapy (RT) had an increased risk of overall SPMs, hematologic malignancies, esophageal cancer, liver cancer, lung cancer, and urinary bladder cancer compared with those who did not receive RT.

**Conclusion:**

Men with PCa tended to have a lower risk of SPMs, but a significantly higher risk of subsequent thyroid cancer. Continued cancer surveillance is required among PCa survivors, especially in specific sites and in individuals who received RT.

## Introduction

Prostate cancer (PCa) is the second most common cancer worldwide in men, with an incidence of 31.1 per 100,000 [1]. PCa prevalence is approaching epidemic levels in the US and Western countries [2]. In the Far East, the incidence of PCa is increasing [3, 4], a trend also observed in Taiwan, where approximately 4800 new cases were diagnosed in 2013, a nearly six-fold increase in incidence compared to 1994 [5]. The increase may result from the widespread adoption of serum prostate-specific antigen (PSA) screening, which has also led to potential reductions in advanced disease and PCa-specific mortality [6]. This technique, combined with advances in surgery, radiation therapy (RT), and androgen deprivation therapy (ADT), have led to increased survival of PCa patients, with current estimates of 10-year and 15-year relative survival at 98% and 91%, respectively [7]. Given the increases in incidence and the life expectancy of PCa patients, there is growing interest in predicting the risk of second primary malignancies (SPMs) among PCa survivors.

Most studies on SPMs after primary PCa in the PSA era were conducted in the US or Europe with inconsistent results [8–12]. The largest Surveillance, Epidemiology, and End Results (SEER) Program-based analysis of 441,504 men with PCa in the US [8] showed a significantly reduced risk of 40% for overall SPMs compared with the general population. However, studies from Italy [10] and France [9] including 4,528 and 3,746 men, respectively, did not observe this reduced trend in SPMs after PCa. Moreover, reports from Switzerland [11] and Germany [12] including 20,559 and 59,259 men, respectively, showed that men with PCa had an overall increased risk of 11–14% of SPMs.

In the Asian population, the risk of SPMs after PCa has not been well documented. A hospital-based series from Japan involving 312 patients found PCa that tended to be associated with a lower risk of developing SPMs, but this difference did not reach statistical significance (relative risk 0.71, 95% confidence interval [CI] 0.45–1.4) [13]. A population-based cohort study from Korea including 55,178 patients found that men with PCa had a significant overall lower risk (25%) of developing SPMs, but it did not record comorbidities other than cancers [14]. Not only the overall risk but also the sites of SPMs development after PCa differ among studies. The discrepancies in these studies could be attributed to therapy for the initial cancer, comorbidities, lifestyles, environmental factors, genetic susceptibility, and ethnic heterogeneity.

The identification of patients or organs at increased risk of SPMs would assist in optimizing treatment for primary PCa and the prompt diagnosis of SPMs. To clarify this issue, we investigated the risk of SPMs in patients with primary PCa in Taiwan, using a population-based cohort study. The relative risk of developing SPMs according to treatment status was also analyzed.

## Methods

### Data source

The data in our retrospective cohort study were obtained from the National Health Insurance Research Database (NHIRD) under the National Health Insurance (NHI) program. The NHI program was established in Taiwan in March 1995, and 99% of the population was enrolled (23.74 million individuals) [15]. The NHIRD has been the source of many epidemiological studies published in peer-reviewed journals [16, 17]. Disease identification was based on the International Classification of Diseases, Ninth Revision, Clinical Modification (ICD-9-CM).

### Ethics statement

The NHIRD encrypts patient personal information to protect privacy and provides researchers with anonymous identification numbers associated with relevant claims information, including sex, date of birth, medical services received, and prescriptions. Therefore, patient consent is not required to access the NHIRD. This study was approved to fulfill the condition for exemption by the Institutional Review Board (IRB) of China Medical University (CMUH104-REC2-115-CR1). The IRB also specifically waived the consent requirement.

### Sampled participants

PCa patients newly diagnosed (ICD-9-CM code 185) between January 1, 2000 and December 31, 2010, from the Registry of Catastrophic Illness Database (RCIPD), a subset of the NHIRD, were defined as the PCa cohort. The date of diagnosis of PCa was defined as the index date. Exclusion criteria included other cancers (ICD-9-CM codes 140–184, 186–208) prior to the index date or age younger than 20 years. Finally, the study included 30,964 patients with newly diagnosed PCa. The non-PCa cohort consisted of patients randomly selected from the Longitudinal Health Insurance Database 2000 (LHID2000) without a history of PCa or other cancers. For each PCa case, we randomly selected one non-PCa control, frequency matching based on age (5-year intervals) and comorbidities of hypertension, hyperlipidemia, diabetes, stroke, heart failure, chronic obstructive pulmonary disease (COPD), coronary artery disease (CAD), alcohol-related illness, and asthma, and index year.

### Outcome and relevant variables

To exclude synchronous primary cancers, SPMs required a minimum 6-month latency period after the primary diagnosis. All study subjects were followed up until they were diagnosed with SPMs (ICD-9-CM 140–184, 186–194, 200–208) or censored because of withdrawal from the NHI program, death, or the end of 2011. Baseline comorbidities included hypertension (ICD-9-CM codes 401–405), hyperlipidemia (ICD-9-CM code 272), diabetes (ICD-9-CM codes 250), stroke (ICD-9-CM codes 430–438), heart failure (ICD-9-CM code 428), COPD (ICD-9-CM codes 491, 492, 496), CAD (ICD-9-CM codes 410–414), alcohol-related illness (ICD-9-CM codes 291, 303, 305, 571.0, 571.1, 571.2, 571.3, 790.3, A215, and V11.3), and asthma (ICD-9-CM code 493). PCa-related treatments (including RT, ADT, and prostatectomy) were also considered.

### Statistical analysis

The Chi-square test was used to examine differences in categorical variables between the PCa and non-PCa cohorts, while the two sample Student’s *t*-tests were used for continuous variables. The follow-up period (in person-years) was used to estimate incidence density rates of SPMs in both cohorts. Univariable and multivariable competing risks regression models were used to estimate subhazard ratios (SHRs) and 99.72% CIs of SPMs development associated with PCa compared with those in the non-PCa cohort. The Bonferroni adjustment was used in multiple comparisons. Multivariable models were also used to investigate the association between PCa and risk of SPMs development, stratified by age and follow-up duration, and the models were adjusted for age and comorbidities. Further analysis was performed to assess the effect of treatment status on the risk of SPMs. Each treatment modality, including RT, ADT, and prostatectomy, prescribed to the study cohort for each patient was calculated independently. All data analyses were performed using SAS for Windows (Version 9.4, SAS Institute Inc., Cary, NC, USA). A two-tailed *P* value < .0028 was considered statistically significant.

## Results

Table 1 shows a comparison of the baseline characteristics of patients with and without PCa. The mean ages of the PCa and non-PCa cohorts were 73.6±8.37 and 73.5±8.44 years, respectively. Patients with PCa had a slightly higher prevalence of hypertension (64.6% vs. 63.3%), hyperlipidemia (29.5% vs. 28.2%), COPD (31.6% vs. 30.6%), and CAD (37.1% vs. 35.7%) than subjects in the non-PCa cohort (*P* values < 0.05). Of the 30,964 PCa patients, 77.4% received ADT, 37.7% underwent RT, and 15.8% underwent prostatectomy. The average follow-up durations were 4.40 ± 3.05 and 5.04 ± 3.25 years for the PCa and non-PCa cohorts, respectively.

**Table 1:** Baseline characteristics in patients with and without prostate cancer.

	Prostate cancer		
	No	Yes	
	N = 30964	N = 30964	
Characteristics	n(%)	n(%)	*p*-value
**Age (years), mean (SD)** ^#^	73.5(8.44)	73.6(8.37)	0.44
**Comorbidity**^†^			
Hypertension	19595(63.3)	19996(64.6)	0.001
Hyperlipidemia	8741(28.2)	9139(29.5)	0.004
Diabetes	5166(16.7)	5299(17.1)	0.15
Stroke	3359(10.9)	3219(10.4)	0.07
Heart failure	2520(8.14)	2549(8.23)	0.67
COPD	9471(30.6)	9772(31.6)	0.01
CAD	11048(35.7)	11473(37.1)	0.004
Alcohol-related illness	1237(3.99)	1227(3.96)	0.84
Asthma	3940(12.7)	4047(13.1)	0.20
**Treatments**^‡^			
Radiotherapy		11672(37.7)	
Androgen deprivation therapy		23961(77.4)	
Prostatectomy		4897(15.8)	

^#^: Two sample T-test;

^†^: Chi-Square Test

^‡^: Patients may have received more than one treatment modality, so the total exceeds the study sample size.

The PCa cohort had a trend of lower SPMs incidence rate (13.5 per 1000 person-years) than the non-PCa cohort (15.1 per 1000 person-years; crude SHR = 0.95, 99.72% CI = 0.89–1.01; Table 2). By multivariable analyses, after controlling for age, hypertension, hyperlipidemia, diabetes, stroke, heart failure, COPD, CAD, alcohol-related illness, and asthma, the PCa cohort had a lower risk for SPMs (adjusted SHR [aSHR] = 0.94, 99.72% CI = 0.89–1.00) than the non-PCa cohort, but the difference did not reach statistical significance. Patients with PCa exhibited a significantly lower risk of liver cancer (aSHR = 0.71, 99.72% CI = 0.60–0.84) and lung cancer (aSHR = 0.72, 99.72% CI = 0.63–0.82) than those without PCa. Conversely, the PCa cohort exhibited a significantly higher risk of thyroid cancer (aSHR = 4.62, 99.72% CI = 2.09–10.2) than the non-PCa cohort.

**Table 2:** Comparison of incidence and subhazard ratios of types of second primary malignancies according to prostate cancer status.

	Prostate cancer		Control			
Site of cancers (ICD-9-CM)	Case	Rate^#^	Case	Rate^#^	Crude SHR (99.72% CI)	Adjusted SHR^†^ (99.72% CI)
All cancers	1831	13.5	2358	15.1	0.95(0.89, 1.01)	0.94(0.89, 1.00)
Hematologic malignancy (200–208)	96	0.71	116	0.74	0.96(0.73, 1.25)	0.95(0.73, 1.24)
Head and neck (140–149, 161)	108	0.79	147	0.94	0.94(0.73, 1.21)	0.94(0.73, 1.21)
Esophagus (150)	40	0.29	67	0.43	0.69(0.47, 1.03)	0.70(0.47, 1.03)
Stomach (151)	173	1.27	189	1.21	1.05(0.85, 1.28)	1.04(0.85, 1.28)
Rectum/anus (153)	280	2.06	280	1.80	1.18(1.00, 1.40)	1.16(0.99, 1.37)
Colon (154)	138	1.01	171	1.10	0.97(0.78, 1.21)	0.98(0.78, 1.22)
Liver (155)	211	1.55	354	2.27	0.72(0.61, 0.85)*	0.71(0.60, 0.84)*
Cholangiocarinoma (156)	24	0.18	36	0.23	0.77(0.46, 1.29)	0.75(0.45, 1.25)
Pancreas (157)	36	0.26	63	0.40	0.65(0.44, 0.98)	0.64(0.42, 0.95)
Lung(162)	343	2.52	562	3.60	0.72(0.63, 0.82)*	0.72(0.63, 0.82)*
Melanoma (172)	7	0.05	8	0.05	0.89(0.33, 2.35)	0.88(0.33, 2.33)
Skin (173)	54	0.40	61	0.39	0.99(0.69, 1.42)	0.98(0.69, 1.41)
Testis (186)	1	0.01	3	0.02	0.48(0.05, 4.74)	0.49(0.05, 5.03)
Urinary bladder (188)	158	1.16	135	0.87	1.37(1.09,1 .72)	1.34(1.07, 1.69)
Kidney (189)	58	0.43	73	0.47	0.95(0.68, 1.35)	0.93(0.66, 1.32)
Brain (191)	20	0.13	16	0.12	0.91(0.47, 1.73)	0.89(0.47, 1.71)
Thyroid (193)	29	0.21	10	0.06	4.88(2.17, 10.9)*	4.62(2.09, 10.2)*
Others	83	0.61	99	0.63	0.99(0.74, 1.33)	0.97(0.72, 1.29)

Rate^#^, incidence rate, per 1,000 person-years; Crude SHR, relative subhazard ratio;

Adjusted SHR^†^, multivariable analysis including age, comorbidities of hypertension, hyperlipidemia, diabetes, stroke, heart failure, COPD, CAD, alcohol-related illness, and asthma

*p<0.0028

Among patients aged ≤ 64 years, those with PCa had a higher risk of thyroid cancer (aSHR = 9.94, 99.72% CI = 2.07–47.8) than those without PCa (Table 3). Among patients aged ≥ 65 years, those with PCa had a lower risk of liver cancer (aSHR = 0.69, 99.72% CI = 0.57–0.83), and lung cancer (aSHR = 0.72, 99.72% CI = 0.63–0.83) than those without PCa.

**Table 3:** Cox proportional subhazards model with subhazard ratios and 99.72% confidence intervals of types of second primary malignancies associated with primary prostate cancer stratified by age and follow-up time.

	Age≤64 years		Age ≥65 years		Follow-up time ≤5 years		Follow-up time >5 years	
	Prostate cancer		Prostate cancer		Prostate cancer		Prostate cancer	
	No(N = 3894)	Yes(N = 4648)	No(N = 27070)	Yes(N = 26316)	No(N = 17666)	Yes(N = 20273)	No(N = 13298)	Yes(N = 10691)
Site of cancers (ICD-9-CM)	Adjusted SHR^†^ (99.72%CI)		Adjusted SHR^†^ (99.72%CI)		Adjusted SHR^†^ (99.72%CI)		Adjusted SHR^†^ (99.72%CI)	
All cancers	1(Reference)	1.07(0.87, 1.33)	1(Reference)	0.93(0.87, 0.99)	1(Reference)	0.79(0.74, 0.85)*	1(Reference)	1.04(0.93, 1.16)
Rectum/anus (153)	1(Reference)	0.76(0.42, 1.38)	1(Reference)	1.22(1.03, 1.45)	1(Reference)	1.00(0.81, 1.22)	1(Reference)	1.22(0.92, 1.62)
Liver (155)	1(Reference)	1.09(0.61, 1.95)	1(Reference)	0.69(0.57, 0.83)*	1(Reference)	0.56(0.46, 0.69)*	1(Reference)	0.91(0.67, 1.25)
Lung (162)	1(Reference)	0.82(0.48, 1.42)	1(Reference)	0.72(0.63, 0.83)*	1(Reference)	0.66(0.57, 0.78)*	1(Reference)	0.64(0.50, 0.82)*
Urinary bladder (188)	1(Reference)	2.39(0.97, 5.93)	1(Reference)	1.32(1.04, 1.67)	1(Reference)	1.04(0.80, 1.36)	1(Reference)	1.70(1.08, 2.66)
Thyroid (193)	1(Reference)	9.94(2.07, 47.8)*	1(Reference)	2.40(1.02, 5.64)	1(Reference)	6.05(2.04, 17.9)*	1(Reference)	2.20(0.66, 7.29)

Adjusted SHR^†^, multivariable analysis including age, comorbidities of hypertension, hyperlipidemia, diabetes, stroke, heart failure, COPD, CAD, alcohol-related illness, and asthma;

*p<0.0028

Among those with a ≤ 5-year follow-up duration, patients with PCa exhibited a significantly lower risk of SPMs, liver cancer, and lung cancer than that shown by individuals without PCa (Table 3). Patients with PCa had a higher risk of thyroid cancer (aSHR = 6.05, 99.72% CI = 2.04–17.9) than those without PCa, among those with ≤ 5 years follow-up. Among those with a > 5-year follow-up duration, patients with PCa had a 0.64-fold (99.72% CI = 0.50–0.82) lower risk of lung cancer than individuals without PCa.

Patients with PCa who underwent RT exhibited a significantly higher risk of overall SPMs, hematologic malignancies, esophageal cancer, liver cancer, lung cancer, and urinary bladder cancer than those without RT (Table 4). The risk of hematologic malignancies and urinary bladder cancer were significantly lower in PCa patients treated with ADT than in those not treated with ADT. Patients with prostatectomy exhibited a significantly lower risk of overall SPMs, hematologic malignancies, liver cancer, and urinary bladder cancer than that exhibited by PCa patients not undergoing prostatectomy. The risk of thyroid cancer was significantly higher in PCa patients treated with prostatectomy than in those who did not undergo prostatectomy.

**Table 4:** Comparison of subhazard ratios of types of second primary malignancies according to treatment status among patients with prostate cancer.

	Radiotherapy		Androgen deprivation therapy		Prostatectomy	
	No	Yes	No	Yes	No	Yes
Site of cancers (ICD-9-CM)	Adjusted SHR^†^ (99.72%CI)	Adjusted SHR^†^ (99.72%CI)	Adjusted SHR^‡^ (99.72%CI)	Adjusted SHR^‡^ (99.72%CI)	Adjusted SHR^§^ (99.72%CI)	Adjusted SHR^§^ (99.72%CI)
All cancers	1(Reference)	2.06(1.80, 2.36)*	1(Reference)	0.91(0.82, 1.01)	1(Reference)	0.82(0.72, 0.93)*
Hematologic malignancy(200–208)	1(Reference)	5.57(3.57, 8.68)*	1(Reference)	0.37(0.25, 0.55)*	1(Reference)	0.42(0.23, 0.78)*
Head and neck (140–149, 161)	1(Reference)	1.19(0.60, 2.38)	1(Reference)	0.99(0.64, 1.56)	1(Reference)	0.71(0.43, 1.15)
Esophagus (150)	1(Reference)	4.10(1.97, 8.52)*	1(Reference)	0.96(0.47, 1.95)	1(Reference)	0.54(0.23, 1.27)
Stomach (151)	1(Reference)	0.43(0.19, 0.97)	1(Reference)	0.77(0.55, 1.06)	1(Reference)	0.59(0.38, 0.90)
Rectum/anus (153)	1(Reference)	1.21(0.79, 1.84)	1(Reference)	0.95(0.73, 1.25)	1(Reference)	0.69(0.50, 0.95)
Colon (154)	1(Reference)	1.35(0.76, 2.40)	1(Reference)	0.96(0.65, 1.42)	1(Reference)	0.66(0.42, 1.05)
Liver (155)	1(Reference)	2.31(1.58, 3.39)*	1(Reference)	1.46(1.03, 2.09)	1(Reference)	0.48(0.31, 0.73)*
Cholangiocarinoma (156)	1(Reference)	0.94(0.22, 4.02)	1(Reference)	0.69(0.29, 1.60)	1(Reference)	0.56(0.19, 1.62)
Pancreas (157)	1(Reference)	1.56(0.55, 4.46)	1(Reference)	1.35(0.60, 3.05)	1(Reference)	1.34(0.68, 2.63)
Lung (162)	1(Reference)	3.33(2.57, 4.32)*	1(Reference)	0.85(0.67, 1.09)	1(Reference)	0.70(0.52, 0.94)
Melanoma (172)	1(Reference)	-	1(Reference)	1.08(0.20, 5.92)	1(Reference)	0.44(0.05, 3.60)
Skin (173)	1(Reference)	-	1(Reference)	0.82(0.46, 1.48)	1(Reference)	0.33(0.13, 0.83)
Testis (186)	1(Reference)	-	1(Reference)	-	1(Reference)	-
Urinary bladder (188)	1(Reference)	2.96(1.97, 4.43)*	1(Reference)	0.59(0.43, 0.82)*	1(Reference)	0.29(0.16, 0.51)*
Kidney (189)	1(Reference)	1.37(0.59, 3.21)	1(Reference)	0.95(0.52, 1.75)	1(Reference)	0.71(0.36, 1.40)
Brain (191)	1(Reference)	0.73(0.10, 5.53)	1(Reference)	1.69(0.46, 6.24)	1(Reference)	1.80(0.67, 4.82)
Thyroid (193)	1(Reference)	0.41(0.06, 2.99)	1(Reference)	0.95(0.40, 2.28)	1(Reference)	3.33(1.63, 6.80)*
Others	1(Reference)	1.66(0.85, 3.23)	1(Reference)	0.76(0.48, 1.23)	1(Reference)	0.61(0.35, 1.08)

Rate^#^, incidence rate, per 1,000 person-years;

^†^: Adjusted SHR was calculated by competing risk regression model adjusted for age, comorbidities of hypertension, hyperlipidemia, diabetes, stroke, heart failure, COPD, CAD, alcohol-related illness, and asthma and treatment of androgen deprivation therapy, and prostatectomy.

^‡^: Adjusted SHR was calculated by competing risk regression model adjusted for age, comorbidities of hypertension, hyperlipidemia, diabetes, stroke, heart failure, COPD, CAD, alcohol-related illness, and asthma and treatment of radiotherapy, and prostatectomy.

^§^: Adjusted SHR was calculated by competing risk regression model adjusted for age, comorbidities of hypertension, hyperlipidemia, diabetes, stroke, heart failure, COPD, CAD, alcohol-related illness, and asthma and treatment of radiotherapy, and androgen deprivation therapy.

*p<0.0028

## Discussion

Previous studies showed a negative association between primary PCa and subsequent SPMs [8, 14, 18–23]. While the reasons for this risk reduction are not entirely clear, they may be related in part to patient age at the time of PCa diagnosis. In Taiwan, more than half of patients with PCa are diagnosed in the advanced stage (stage III or IV) [5]. Therefore, older men with PCa, particularly men with competing risks for death, may not have the same opportunity for a second diagnosis as others of the same age. In our study, the cumulative mortality over 13 years (1998–2010) was 32.1% in the PCa cohort, which was much higher than that in the non-PCa cohort (20.7%). In order to better account for the fact that individuals who die do not have the opportunity to develop a second primary malignancy, we use a competing risks model to analyze the data in a population-based cohort. Furthermore, we adjusted for possible comorbidities and found that patients with PCa tended to have a lower overall risk of SPMs (adjusted SHR = 0.94, 99.72% CI = 0.89–1.00, p = 0.06). The main risk reduction in the development of SPMs was noted in the lung and liver. This decrease could be partly explained by reduced medical surveillance in elderly men with PCa.

However, our study showed that men with PCa had a higher risk of SPMs in the thyroid compared to those without PCa. There is a trend for a higher risk of developing SPMs in the urinary bladder (adjusted SHR = 1.34, 99.72% CI = 1.07–1.69, p = 0.015) and rectum/anus (adjusted SHR = 1.16, 99.72% CI = 0.99–1.37, p = 0.114). The SEER Program [8] and other large population-based studies [10, 11, 12, 14] reported that men with PCa had a 5%–109% increase in the relative risk of urinary bladder cancer compared to the general population. Elevated risk of SPMs in the kidney was also noted by some authors [8, 9, 11, 12]; in contrast, this finding was absent in this study and some other studies as well [10, 14]. The significant increase in urinary system cancer may be due to a shared etiology, such as a common carcinogenic pathway related to urinary stasis, chronic inflammatory substances, or genetic mutations [24], particularly for the urinary bladder, which is adjacent to the prostate. It may be argued that detection bias cannot be excluded because the significantly increased SPMs risk in the urinary tract was only noted at the beginning of follow-up [14, 25]. However, we found that even after the 5-year follow-up period, men with PCa had a trend of higher risk of urinary bladder cancer than those without PCa (adjusted SHR = 1.70, 99.72% CI = 1.08–2.66, p = 0.021), suggesting that detection bias is less likely.

The association between PCa and subsequent rectal or colon cancer remains controversial. Cassetti et al. [10] found a significantly increased risk of rectal cancer following a diagnosis of PCa, whereas Heard et al. [26] found an increased risk in both the colon and the rectum. However, no significantly altered SPMs risk in the colon and rectum was demonstrated by Braisch et al. [12] or Levi et al. [23]. Furthermore, the SEER Program and a Korean study noted a significantly reduced risk of SPMs in the rectum after PCa [8, 14], whereas both studies found that patients who received RT had an increased risk of subsequent rectal cancer. Exposure to ionizing radiation is generally considered a risk factor for cancer. A recent systematic review by Wallis et al. reported that RT for PCa was associated with a higher risk of SPMs of the urinary bladder, colon, and rectum compared with no RT or with surgery [27]. We found that men who received RT had an increase in risks of overall SPMs and urinary bladder cancer compared with those who did not receive RT; however, the increased risk was not noted in the rectum. Except the possibility of radiation-induced carcinogenesis, patients treated with RT are often older with more medical co-morbidities, therefore likely more prone to having symptoms associated with urinary retention possibly increasing their risk of bladder cancer. Two possible reasons for the discrepant findings regarding SPMs in the rectum after RT for PCa have been suggested.

First, the SEER and Korean studies collected patients across periods covering almost 20 years, and radiation techniques improved over time (from two-dimensional RT to three-dimensional conformal and intensity-modulated RT) [8, 14]. Our study involved patients who underwent RT more recently, between 2000 and 2010. With increased use of conformal RT, there is a lower radiation dose to critical organs such as the rectum, with correspondingly fewer side effects and SPMs.

Second, the latency period between radiation exposure and a radiation-induced malignancy is estimated to be more than 5 years, ranging up to 15 years. The systematic review of RT-related SPMs for PCa, mentioned previously, indicated that odds ratios for rectal cancer increased with a longer lag time (odds ratio at 5-year lag vs. 10-year lag: 1.68 vs. 2.2) [27]. Furthermore, the SEER Program only considered subsequent primary cancers that developed ≥ 10 years after RT for PCa [8]. In our study, the relatively short follow-up period (mean less than 5 years) among those who received RT could have obscured a significant association between RT and the risk of rectal cancer.

According to our results, men with PCa have an elevated risk of developing thyroid cancer, and this risk was higher in patients diagnosed at younger than 64 years old. The German and Korean studies noted a similar increased risk of SPMs in the thyroid after PCa [12, 14]. In the US, there is a significant association between diagnosis with PCa and thyroid cancer [28]. Several hypotheses can explain the positive association between two cancer diagnoses, such as environmental or genetic risk factors. For instance, the possibility of shared genetic risk factors related to mutations in the proto-oncogene has been reported in both cancer types [29]. However, as the adjusted HR was significantly increased only within a follow-up of less than 5 years, detection bias cannot be excluded to explain the increased SPMs in the thyroid. Although the significantly increased risk of SPMs in the thyroid was noted, the overall number of cases of thyroid cancer was small, particularly in men whose age ≤ 64 years old (13 patients in the PCa group; 1 patient in the control group). It likely explains the extremely wide confidence interval in the analysis restricted to age ≤ 64 years old.

There are limitations to our findings. First, the NHIRD does not contain detailed information on potential confounding factors, such as diet, smoking, and alcohol consumption. We only included comorbidities relevant to the NHIRD in Taiwan. We used smoking-related comorbidities (hypertension, stroke, COPD, CAD, and asthma), alcohol-related illness, and diet-related comorbidities (diabetes and hyperlipidemia) as indicators for smoking, alcohol consumption, and dietary habits, respectively. Second, because there is no link between the NHIRD and the cancer registry, we did not have access to information regarding PCa stage or grade. Furthermore, the NHIRD provided no information on RT dose level, energy level and distribution. This precluded us from performing any analysis involving these variables. Third, the overall duration of the present study was 11 years, shorter than those of previous studies [8, 11]. Longer follow-up periods are necessary to examine the real effect of RT-induced malignancies. Nevertheless, this study is strengthened by a large patient population contributing 136,241 person-years at risk in Taiwan, which is sufficient for valid analyses. In addition, the results were adjusted for possible confounding factors, including age and comorbidities.

## Conclusion

In conclusion, the current analysis showed that patients with PCa tended to have a decreased risk of overall SPMs compared to patients without PCa, but the difference did not reach statistical significance. The main SPMs risk reduction was found in the lung and liver. In contrast, an increased risk remained in PCa patients for the thyroid cancer. There is a trend for a higher risk of developing SPMs in the urinary bladder and rectum/anus. Although the short follow-up period may underestimate RT-related carcinogenesis, we found that PCa patients undergoing RT had a higher risk of overall SPMs than those not undergoing RT. Knowledge of risk factors can contribute to a better understanding of the risk of SPMs and highlight the need for continued cancer surveillance among PCa survivors.

## Supporting information

S1 STROBE Checklist Checklist of items that should be included in reports of observational studies. (DOC)

## Abbreviations

ADT: androgen deprivation therapy
CI: confidence interval
HR: hazard ratio
ICD-9-CM: International Classification of Diseases, Ninth Revision, Clinical Modification
NHIRD: National Health Insurance Research Database
PCa: prostate cancer
PSA: prostate-specific antigen
RT: radiation therapy
SPMs: second primary malignancies
